# On Diminution of the Size of the Child by Regimen and Venesection

**Published:** 1850-03

**Authors:** 


*On Diminution of the size of the Child by Regimen and Venesection.* By M. Depaul.—M. Depaul, fully appreciating the advantages derivable from the induction of premature labor in contracted pelvis, yet believes that the necessity of doing this may be avoided in certain cases, by submitting the mother to regimen and repeated venesection. His attention was first directed to the subject by hearing M. Moreau, in his lectures, express a favorable opinion upon the feasibility of the procedure, founded upon a case that had occurred to him. M. Moreau had attended a woman in two labors, which were very difficult ones, in consequence of considerable pelvic contraction. Apprised that she had again become pregnant, he submitted her to a severe regimen and repeated venesection, and the result was, that a small living infant passed without any aid. In a later pregnancy, she was not submitted to any regimen, embryotomy was had recourse to, and both mother and child died. M. Moreau has since met with other similar cases; but he believes that when the antero-posterior diameter of the pelvis is less than 3¼ inches (French), or, at most, 3 inches, regimen can only be relied upon as an auxiliary. The influence of feeding on the size of the young, is well known in rural districts and to veterinarians; while in women who reject nearly all their food during pregnancy, the child is often very small. This is also the case with the women that come to the *Maternité* to be confined, after undergoing the greatest privations during pregnancy.

Dr. Depaul relates two cases which have occurred in his own practice. Madame G——, of small stature, and having some degree of contraction of the pelvis from rickets, bore, in 1843, a full-sized, still-born child, after a very difficult labor, and the use of ergot. In 1846, she was delivered of a large, still born child by the forceps. She conceived again, November 21st, 1847; and although the induction of premature labor had been predetermined upon in the event of another pregnancy, yet she earnestly entreated that a full trial should be given to regimen. Accordingly, from February, her diet was limited to *potages* and vegetables, a little meat once a week, and half a pound of bread per diem; and although a healthy person, with vigorous appetite, she cheerfully submitted to the privation. About 400 grammes of blood were abstracted from her arm in the 3d, 6th, 8th, and 8½th month. She became pale and thin. On the 19th of Augnst, labor commenced, and was rapidly finished. The child was small, weighing only 5lbs. (the two others had each weighed 9lbs.), but cried and did well. The mother now allowing herself a better diet, soon recovered her strength.

The subject of the *second* case had been formerly attended by M. P. Dubois, who, finding a rickety pelvis, contracted to 3 inches, had perforated the child's head. On the present occasion he had recommended the induction of premature labor at the 8th month. Labor-pains, however, set in of their own accord, and the author, summoned in the absence of M. Dubois, brought away a small, living child, by means of the forceps. It weighed 2600 grammes, and the bones of its skull were of remarkable thinness. It was discovered afterwards, that this woman had really gone her full time, having, after various futile attempts at procuring abortion at an early stage, resolved to submit herself to a debilitating regimen. This she commenced at the second month, being then rather stout, and having an excellent appetite; and she throughout continued an almost starvation regimen. At first she was much tormented by epigastric pain and intense thirst, requiring large quantities of water to allay it, and she soon became very thin and feeble; but having become accustomed to her privation, she suffered, during the last three months but little from its continuance. After her confinement, a judicious diet soon restored her strength and flesh, and she was able to suckle her child. This case is therefore more satisfactory than the former, inasmuch as the same end was obtained without resorting to venesection.

The procedure would be justifiable in those cases in which the woman, without having any notable diminution of the pelvis, yet always brings forth, owing to their size, only dead children. In moderate contraction of the pelvis, too, it may be resorted to, and although not available in cases of excessive contraction, it may even here be used as an auxiliary, to delay somewhat longer the period of inducing premature labor, and thus give the child an additional chance of life. During the progress of the case, frequent examination should be made, so that we may ascertain, by the degree of development of the uterus, the mobility of the child, and the abundance of the liq. amnii, how far our success is probable. If from this, we conclude our end cannot be gained, we should resort to artificial means for terminating the pregnancy. It is not desirable to commence the debilitating regimen, prior to the 3d or 4th month, as before that period the development of the fœtus takes place very slowly, and the pregnancy is much more easily endangered by whatever affects the maternal system. It would, too, be probably better, if the amount of aliments were gradually diminished, so as the more easily to accommodate the system to so painful and so prolonged an experiment. The strong meats should be quite abstained from, and, as a general rule, three fourths of the nutriment should be cut off. Much will depend upon the state of the circulation in guiding our decision as to the employment of venesection.—*Bull. de Thérapeutique, from Brit. and For. Med. Chir. Rev.*

[When we bear in mind, that women who have suffered during their entire pregnancy from excessive sickness (M. Depaul, however, states that some of these do not reject their aliments), and others, whose system has become exhausted by want or disease, yet sometimes bring forth voluminous children, it is evident that M. Depaul's recommendation does not admit of a large generalization. Indeed, with some women, the production of large children seems to be as natural as the possession of any personal peculiarity, and by no means dependent upon the mode of life they adopt. Still, there are cases of deformed pelvis, where it may be of great importance to have a living child, in which the plan is well worthy of a trial; for if, by its agency, a small, living child is born at its full time, its chances of surviving will, doubtless, be very superior to those even of a larger infant prematurely brought forth.]—*Brit. and For. Med. Chir. Rev.*

